# Validation and utility of the French version of the Unified Multidimensional Calling Scale (UMCS-22) for stipended volunteer firefighters

**DOI:** 10.1371/journal.pone.0350184

**Published:** 2026-05-28

**Authors:** Marina Burakova

**Affiliations:** 1 Aix Marseille Univ, LPS, Aix-en-Provence, France; 2 Laboratoire de Psychologie Sociale UR849, Aix-Marseille Université, Aix-en-Provence, France; University of Padua: Universita degli Studi di Padova, ITALY

## Abstract

This study evaluated the psychometric properties of the French version of the Unified Multidimensional Calling Scale (UMCS-22), adapted for use with volunteer firefighters, including reliability, construct and criterion validity. Data were collected electronically via LimeSurvey (N = 1,149) and analysed using Jamovi 2.3.3. The measurement model of the French UMCS-22, examined globally and with respect to measurement invariance across gender and age groups, exhibited strict invariance. Internal consistency coefficients ω-McDonald of the dimensional scores of the French UMCS-22 fell between.75 and.86; while its global score had ω = .96. The evaluation of criterion validity through Bayesian linear regression and Structural equation modelling demonstrated differential functioning of the French UMCS-22 facets. Specifically, transcendent summons and pervasiveness were associated with the negative outcomes and purposefulness did not predict any outcome. The utility of the French UMCS-22 adapted for stipended volunteer firefighters is discussed with regard to the neoclassical and modern approaches as well as with regard to the double-edged sword nature of calling.

## Theoretical background

### Nature and dimensionality of calling

The construct of occupational calling has been explored through various conceptual frameworks that converge on several core elements, including a sense of meaning, purpose, and an orientation toward others [1–5]. Calling is directed toward a specific domain rather than work in general [6], and can be experienced in two forms: (a) as a perceived calling, denoting the feeling of being summoned to a particular occupation, and (b) as a lived calling, representing the realization of a superordinate goal and the generation of a tangible social contribution [3,4]. Consequently, calling is characterized by both stability and dynamism. Its stability stems from higher-order goals, particularly reflected in its transcendent, prosocial, and purposeful dimensions. Thus, it should be distinguished from constructs such as flow, and work engagement, which are more transient and short-term in nature [5,6]. Its dynamic, or developmental, aspect is associated with goal pursuit [2,4,5]. In a longitudinal study, Dobrow and Tosti-Kharas [2] reported strong correlations of calling scores over a six-week interval (r = .83), and moderate correlations over 3.5-year (*r* = .41) and 7-year (*r* = .38) periods. While calling was initially viewed as a source of life meaning and satisfaction [6], more recent literature has highlighted its potential drawbacks, such as associations with workaholism and burnout, thus framing it as a “double-edged sword” [3]. This has prompted calls for a more nuanced exploration of its dimensions and outcomes [1,5].

Two major theoretical perspectives have informed the understanding of the nature and dimensionality of calling: the neoclassical and the modern approaches. The neoclassical view conceptualizes calling as externally driven, focused on the notions of social utility, meaningfulness, sense of duty, and mission, and therefore associated with eudemonic well-being [3,5–9]. In contrast, the modern perspective posits calling as internally driven, not necessarily producing socially useful outcomes, but rather motivated by self-actualization, pursuit of personal happiness, and linked with hedonic well-being [2,8,9]. At the dimensional level, transcendent summons, prosocial engagement, and sacrifice would be rather associated with the neoclassic approach. Transcendent summons refers to guidance originating beyond the self, such as a higher force or inner voice that offers direction and certainty [1,10]; sacrifice – to a sense of duty [2,6], and prosocial orientation – to altruistic behavior [5]. Passion and identity would be more often addressed with regard to the inner focus of calling, and, thus, to the modern conceptualization of calling. Passion is linked to enjoyment and pursuit of personal happiness [2,7]. Identity refers to a sense of ownership over one’s occupation [10]. Purposefulness may be associated with either neoclassic [1,6] or modern approach [2,5], while being stressed as a core aspect of calling that distinguishes it from other occupation-related constructs. It denotes personal significance of one’s work and its contribution to life meaning, as well as the congruence between work and personal values [5,10]. Pervasiveness is associated with cognitive ruminations and boundary-blurring tendencies and with further negative health outcomes, such as burnout at work [8,9]. While calling dimensions are clearly conceptualized and distinguished at the conceptual level, they seem to be tightly connected and nourishing each other in reality. For example, Bunderson and Thomson [7] reveal through the narratives of the zoo-keepers how the transcendent aspect of calling grounded the identification with the occupation and the construction of its meaning.

### Measurement of calling

Several instruments have been developed in alignment with these conceptual frameworks. The neoclassical approach inspired the creation of three unidimensional and two multidimensional measures. Among the unidimensional tools, the earliest was the *Pennsylvania University Work-Life Questionnaire* [6], comprising eight items contrasting calling with job and career orientations, validated among 238 university employees. Treadgold [11] developed the *Engagement in Meaningful Work Scale*, which aimed to assess intrinsic work motivation and inner guidance, though item specifics were not disclosed; this scale was validated with 127 participants examining associations with depression, stress, and self-concept. Dreher et al. [12] introduced the *Vocation Identity Questionnaire (VIQ)*, a nine-item instrument based on the Reformation-related dimensions such as intrinsic motivation and perceived significance, validated on a university staff sample. Bunderson and Thompson [7] created a six-item *Neoclassical Calling Questionnaire* for zookeepers, incorporating dimensions such as passion, transcendent summons, and purposefulness, with job and organizational attitudes examined as outcomes in a sample of 775 individuals. Two multidimensional measures reflect the neoclassical perspective. Hagmaier and Abele [10] developed the *Multidimensional Measure of Calling*, consisting of nine items measuring identification, sense and meaning, and a transcendent guiding force, validated on 211 working adults. Dik et al. [1] introduced the *Calling and Vocation Questionnaire (CVQ)*, a 24-item instrument covering purposeful work, transcendent summons, and prosocial orientation, operationalized within “presence” and “search” modes, and validated with 456 undergraduate students.

Within the modern framework, two major instruments are notable. Dobrow and Tosti-Kharas [2] proposed a 12-item unidimensional *Calling Scale* assessing passion, identity, urgency, sacrifice, and pervasiveness, validated on a sample of 1,500 working adults (a part of whom participated in a longitudinal assessment) with various job outcomes, including job involvement and work engagement. Praskova et al. [5] developed the multidimensional *Career Calling Scale for Emerging Adults*, validated on 527 students and comprising three dimensions: other-oriented meaning, personal-oriented meaning, and active engagement, with life satisfaction as a criterion variable.

Of these instruments, five are unidimensional [2,6,7,11,12], and three are multidimensional [1,5,10]. In their meta-analysis, Dobrow and Tosti-Kharas [8] emphasize that although these instruments are conceptually aligned with either the neoclassical or the modern perspective on calling, they may nonetheless partially overlap with the alternative conceptualization. Given the inability of unidimensional measures to capture the complexity of calling and the partial coverage of dimensions in multidimensional instruments rooted in either framework, Vianello et al. [9] developed a new multidimensional instrument – the Unified Multidimensional Calling Scale (UMCS-22). The UMCS-22 assesses seven dimensions: passion, sacrifice, transcendent summons, prosocial orientation, pervasiveness, purposefulness, and identity. The 22-item scale was validated across three waves involving a large sample of Italian university students: 5,886 in the first wave, 1,700 in the second, and 881 in the third, with 434 participants completing all three waves. The scale demonstrated good model fit overall, although item 3 of the pervasiveness subscale loaded poorly (*β* = .30). The UMCS-22 thus represents a robust instrument with the most comprehensive coverage of the theoretical dimensions of calling.

### Significance of calling for volunteer work

Calling represents a promising research perspective with regard to its outcomes within professional and paraprofessional activities [e.g., 13–15]. According to the recent developments in the Job Demands-Resources (JD-R) theory [16], it appears among the key psychological resources protecting individuals from health impairments. At the same time, certain voices advocate a more nuanced interpretation of calling outcomes hypothesizing its association with overengagement, workaholism, and burnout [e.g., 3,17]. Yet, comparative or systematic evidence of the effects of different facets of calling remains scarce. Indeed, most empirical studies report the associations between global calling and its outcomes without distinguishing calling dimensions [e.g., 4,14,18–20]. An overall conclusion of such studies consists in considering calling as increasing job and life satisfaction, thanks to its capacity to transform challenging job demands into work engagement and job satisfaction as well as a protective factor against burnout and turnover [16]. Fewer publications focus on the relationships between calling dimensions and outcomes [5,8–10].

Two issues warrant clarification: (1) whether calling dimensions associated with the neoclassical and modern approaches differ in their relative salience, and (2) which dimensions are linked to the potential “dark side” of calling, leading to occupational health impairments and other negative outcomes. Thus, recent meta-analytic findings [8] indicate that the eudaimonic, or externally oriented, dimension of calling – aligned with the neoclassical framework – has a stronger positive impact on the perceived meaningfulness of work than its hedonic, internally oriented, counterpart associated with the modern approach (*rs* = .80 versus.58). Conversely, hedonic outcomes, such as job satisfaction, are better explained by internally drive aspects of calling (*rs* = .47 versus.38). With respect to broader positive outcomes, such as psychological well- being, stronger associations are again observed with external aspects of calling (*rs* = .49 versus.37) [8]. At the same time, in the validation study of the UMCS-22 on a student sample [9], passion, which is internally oriented, exhibits the strongest positive effects with the outcomes, including satisfaction with studies [*ß* = .62] and intention to pursue the study programme [*ß* = −.17], followed by purposefulness [*ß* = .09 and.10] and sacrifice [*ß* = .08 and.02]. The discrepancies between the effects reported for eudaimonic (neoclassical approach) and hedonic (modern approach) dimensions in Dobrow et al. [8] and Vianello et al. [9] may reflect differences in measurement instruments and samples. To recall, the UMCS-22 [9] is the only multidimensional scale encompassing both conceptualizations. As regards the dark side of calling, it has received increasing attention in recent literature challenging its initial positive conceptualization [3,7–10,15]. Some researchers [e.g., 3, 15] suppose that the dark side of calling may emerge from the interaction between calling and certain dispositional characteristics and organizational contexts. For others [e.g., 7], a very strong neoclassical calling may heighten vulnerability to organizational exploitation, as employees may accept sacrificing income, time, and well-being for their work. Negative effects of certain calling dimensions are reported in the UMCS-22 validation study [9]. Prosocial orientation and identity exhibit negative effects with satisfaction with studies [*ß* = −.05 and −.07]; transcendent summons and pervasiveness have negative, albeit nonsignificant, effects on satisfaction with studies [*ß* = −.04 and −.06] and intention to pursue the study programme [*ß* = −.05; *ß* = −.05] in Italian students [9]. Beyond a few findings reporting magnitudes and directions of the effects of different calling dimensions [8,9], the differential functioning of the facets of calling remains insufficiently specified and warrants further investigation.

As regards the target populations addressed by the growing cluster of calling research, educators and students remain its primary focus [e.g., 1,5,6,9,20]. At the same time, volunteer workers, who incarnate calling while being driven by the motives of altruism, social utility, self-actualization, and passion [21–23], remain under considered [8]. Also called “stipended volunteers”, volunteer workers are different from unpaid volunteers: they are formally engaged with their respective organizations in addition to their main occupation and receive a gratification in exchange for the provided service [e.g., 24,25]. Given a less constraining form of contract, they may easily quit their organizations, yet they represent an important human resource for the civil security and emergency service in many countries, including Australia, Germany, Finland, France, United States, etc. [e.g., 26,27]. Emerging research suggests that calling plays a central role in linking volunteers to their organizations, thereby predicting their satisfaction and retention [22]. For individuals, who do not derive purpose from their occupations, volunteer activities may provide a compensatory sense of life meaning [22]. At the organizational level, instruments measuring calling can serve as valuable tools for refining human resource practices.

To summarize, the issues of measurement and outcomes of calling seem to be interrelated. The UMCS-22 [9] is the only multidimensional instrument reconciling the neoclassical and modern approaches. The recent works suggest that, contrary to the previous believes, calling might have its dark side and, therefore, lead to the negative outcomes [3,16]. Beyond a few findings [8,9], the differential functioning of the facets of calling remains yet to specify. Students and employees of the educational organizations remain the main targeted population of the calling research, while volunteers exhibiting high levels of calling are still understudied. Therefore, the present study aimed at the validation in French of the UMCS-22 among French stipended volunteer firefighters (VFF). This included the assessments of construct and criterion validity, as well as of reliability of the scale and subscale scores. To extend the understanding of the differentiated functionating of calling facets and to contrast dimensions aligned with the neoclassical and modern frameworks, the French UMCS-22 dimensional scores were examined in relation to key outcome variables relevant to VFF: emotional exhaustion, job satisfaction and turnover intention [21,26].

## Method

### Analytic strategy

1 The UMCS-22 [9] was translated into French following the cross-cultural construct equivalence method [28], with the involvement of a panel comprising three bilingual experts and a pretest panel composed of 10 representatives of the target population (S1, Tables in S1 File).
  a One item originally reverse-worded in the scale was rephrased in a direct mode for the French version (item Per_3), in accordance with recent psychometric recommendations advising against the combination of direct and reverse wordings in languages with complex syntactic negation structures [29, 30]. Specifically, an item from the pervasiveness subscale (*My days would be less meaningful if I was not involved in these studies* (English*)/Le mie giornate avrebbero meno senso se non stessi facendo questi studi* (Italian)) was identified as reverse-worded and previously showed a low factor loading (.28) [9]. Its direction was changed (*Mes journées ont du sens grâce à mon activité de sapeur-pompier volontaire* (French)/*My days are meaningful thanks to the volunteering firefighting* (English)). Otherwise, item wording was as close as possible in terms of the principles of the culturally contextualized adaptation. As regards the adaptation of the items to the context of volunteer firefighting, given that calling is not an abstract construct but is instead directed toward a specific target activity [6], the term “volunteer firefighting activity” was used in place of “studies” in the original scale [9]. (S1, Tables in S1 File).
  b A pretest of the French version of the UMCS-22 was conducted with a panel of 10 volunteer firefighters (*M*_*age*_ = 41.10; *SD*_*age*_ = 11.27; *M*_*length of service*_ = 15.20; *SD*_*length of service*_ = 8.18). The panel assessed the items of the French UMCS-22, which lead to the reformulation of four items perceived as impersonal. These revisions involved replacing impersonal grammatical structures with first-person constructions using the pronoun I. The adjustments concerned one item from the *Passion* subscale, two items from the *Prosocial orientation* subscale, and one item from the *Pervasiveness* subscale (S1, Tables in S1 File). To provide an example, in the item from the *Prosocial orientation* subscale “Helping others is my primary motivation in my career” (item “Pro_3”), the expression “helping others” was replaced by “bettering others’ lives” (S1, Tables in S1 File).
  c In line with the established recommendations for scale development and validation [31,32], the panel also evaluated the face validity of the items measuring the outcome variables, as well as the length of the response scale. Regarding face validity, items should be perceived as meaningful and relevant, easy to answer, unambiguous, nonjudgmental, and not overly sensitive. With respect to the rating format, odd-numbered scales allow for a midpoint, which may function as an escape or non-response option, whereas even-numbered scales, when combined with a non-mandatory response format, are associated with more informative missing data, which may indicate participant withdrawal [e.g., 33]. Current methodological recommendations generally favor 5- or 6-point Likert scales, with 4-point scales considered a lower bound and 7-point scales an upper bound in terms of discriminant validity and reliability [e.g., 34,35]. In addition, fully labeled response scales have been shown to provide more precise data than scales with labeled poles only [36], and shorter scales are associated with lower cognitive load when processing response options [34]. Accordingly, three fully labeled response scale lengths were evaluated by the target population panel: 4-, 5-, and 6-point scales. The 4-point scale received the highest ratings for all three outcome variables: satisfaction with firefighting (M = 4.90 vs. 4.10 and 3.20, respectively), exhaustion (M = 4.90 vs. 3.90 and 3.10, respectively), and turnover intention (M = 4.70 vs. 3.60 and 3.10, respectively).
2 To assess the criterion validity of UMCS-22 scores, three items capturing key outcomes relevant to volunteer firefighters (VFF) were included: emotional exhaustion, job satisfaction, and turnover intention [21]. The choice and the relevance of the items are specified in the Measures section. All study variables were measured using a 4-point Likert scale, selected for improved usability based on the pretest results (Step 1b).
3 A minimum sample size was calculated via the online calculator designed on the basis of the formula suggested by Kim [37]. For the measurement model including 7 latent variables, expected average factor loadings of.60, CFI = .95, p < .05, factor r = .30, ß = .90 (power), dropout rate = 10% was of 536 participants [37].
4 The protocol designed in accordance with Helsinki Declaration was approved by the ethical committee of Aix-Marseille University. An informed consent should be obtained electronically after the detailed description of the study and data management procedure and prior to the enrolment into the survey. With regard of the sensitive character of the data (VFF are actors of the French civil security system), a cross-sectional design was adopted and a very limited range of sociodemographic characteristics was captured.
5 The survey (S1, Tables in S1 File), hosted on LimeSurvey, was administered electronically nationwide via the advertisement published by the French Federation of Firefighters and on the intranet of a single Fire and Rescue Service in the South of France. None significant difference is observed in the subsamples’ scores. The anonymized data used for this validation study are available at: https://osf.io/7jqtg/files/jfn3p
6 Data analysis was performed using Jamovi (version 2.3.3), selected for open-access availability and broad coverage of contemporary statistical techniques. In particular, probabilistic estimation methods based on Bayes factor were favored for their ability to provide more stable and informative parameter estimates [38–40]. These methods are less sensitive to violations of normality assumptions, especially for interval-level variables, and explicitly account for measurement error. In addition, Bayesian techniques allow for the quantification of evidence in favor of both the alternative hypothesis (H1) and the null hypothesis (H0), while permitting the assumption of equal prior probabilities for either hypothesis. This approach aligns with the ATOM principle (**A**ccept uncertainty. Be **t**houghtful, **o**pen, and **m**odest.) formulated by Wasserstein et al. [41, p. 2] which advocates moving beyond the reliance on *p*-value in research. The following thresholds are recommended for the evidence assessment: 1 < BF_10_ < 3 – weak evidence; BF_10_ ≥ 3 – moderate evidence; BF_10_ ≥ 10 – strong evidence; BF_10_ ≥ 30 – very strong evidence; BF_10_ ≥ 100 – extreme evidence [39].
  a *Outliers’* management was conducted via many-facet Rasch model (MFRM, module snowIRT) [41]. The mean-square (MS) values to be retained for further analysis should fall within the interval [.05; 1.5] [42].
  b *Construct Validity*, understood as the extent to which the French UMCS-22 reflects the theoretical construct of calling [43], was evaluated by assessing the overall quality of the measurement model according to established thresholds [44]. The method of weighted least squares with mean and variance adjustment (WLSMV) was chosen to assess the model due to the use of the 4-point Likert scale) [40]. Configural, metric, scalar, and strict invariance were tested across samples, gender, and age groups. Non-invariance was considered negligible when the RMSEA difference was < .015 and the CFI change was < .010 [45]. Cross-sample measurement invariance was based on the principle of generalizability [32]. The test of gender measurement invariance was informed by social role theory [46]. In the population under study, gender asymmetry remains relatively stable across countries, with women rarely exceeding 20% of the workforce [e.g., 47]. Although evidence exists for physiological and cognitive differences between male and female firefighters, such as lifting capacity and reaction time [e.g., 48], research suggests that the primary barriers to gender equity are organizational and cultural rather than individual [e.g., 49]. The assessment of age-related measurement invariance was theoretically grounded in life-span developmental theory that considers preferences as evolving across the life course [50], and empirically informed by meta-analytic findings suggesting that calling tends to decline with age [8]. Age groups were defined in line with the French legislation and informed by national statistical classifications. In France, the National Institute of Statistics and Economic Research (INSEE) distinguishes three age cohorts: 15−24, 25−49, and 50−65 years [51,52]. This classification is based on both legal thresholds and socio-psychological analyses of age and employment. The French legislation establishes the minimum legal age for employment at 16 years, with limited exceptions at 15 years (e.g., apprenticeships and seasonal employment), and sets the legal retirement age at 64 years, with possible variations between 62 and 67 years depending on birth cohort [53,54]. Occupational trajectories in France vary across age groups with respect to self-regulation, the balance between demands and resources, and organizational identification [e.g., 55]. Accordingly, the early career stage (16−24 years) is characterized by a limited control over work activity; the mid-career stage (25−49 years) represents the organizational reference model; and the late career stage (50−65 years) places greater emphasis on the sustainability of work activity. From this perspective, age is understood as an indicator of position within the work system rather than as a biological variable [55]. The capacity of the French UMCS-22 to cover all relevant aspects of calling [43] was assessed through item factor loadings, with values ≥ .70 considered strong and ≥.50 acceptable [56].
  c Given the multidimensional and context-specific nature of the instrument, *Reliability* was estimated using McDonald’s omega (ω) recommended for congeneric scales [57–59]. Reliability may be interpreted as excellent when ω ranged from.80 to.90, and good between.70 and.80 [59]; ω ≥ .90 (CI = 95%) as aligning with higher stakes standards and ω ≥ .65 (CI = 95%) as aligning with lower stakes standards [58].
  d *Convergent Validity* at the construct level was assessed via the indicator AVE (average variance extracted) that reflects the average of all squared factor loadings. It is considered as good when AVE ≥ .70 and acceptable when AVE ≥ .65 [57].
  e *Criterion Validity*, defined as the ability of the instrument to predict relevant outcomes [31,43], was evaluated via methodological triangulation, including three methods – Bayesian correlation, Bayesian linear regression, and SEM – addressing the relationships between the French UMCS-22 scores and key criteria (job satisfaction, emotional exhaustion, and turnover intention). Bayesian linear regression (JSQ module) was employed to estimate the probability, direction, and effect size of predictions. As previously stated, this method offers an advantage over traditional frequentist approaches by assessing the best fitting model in terms of outcome representation, beyond mere statistical significance [38,39]. The thresholds for Bayes factor interpretation are specified above (see point 6).
  f To inform about the relationship between the UMCS-22 dimensional and global scores with the criteria and numeric sociodemographic variables, including age and service length, Bayes Pearson correlations were calculated. For nominal sociodemographic variables, including sex and ranks, Bayes T-test and Bayes ANOVA were performed.

### Measures

Firefighters operate under intense time pressure and demanding schedules, with operational patterns that limit opportunities for recovery and rest and contribute to work-life imbalance and reduced available spare time [21,60]. These constraints, together with feedback from the target population, motivated the use of single-item measures derived from the scales previously used within the target population [21,61]. Single-item measures are less time-consuming and are often perceived as less redundant by respondents, which may reduce non-response and survey break-off while maintaining acceptable levels of validity compared to multi-item measures [e.g., 31, 62]. Beyond decreasing cognitive burden, single-item measures may also decrease criterion contamination and better face validity of a scale [63].

Indeed, Fisher and colleagues [63], advocated for the existence of substantial evidence in favor of single-item measures related to concrete unidimensional and semantically clear constructs, such as job satisfaction, occupational stress, social support, job insecurity, bullying in the workplace, etc. Moreover, they provided findings supporting the validity and reliability (assessed via the communality index) of 18 single-item measures among which job satisfaction and emotional exhaustion. Thus, a single-item measure of satisfaction (*Overall, I am satisfied with my job*) showed acceptable communality index (*h*^*2*^ = .76) and acceptable test-retest reliability at an 18-month lag (r_tt_ = .70/.60). Another study [64] supported the robustness of a single-item measure extracted from the French version of the SWLS-W. Cheung and Lucas [65] reported similar results for a single-item measure extracted from the SWLS across two North-Americal and one German samples.

The burnout single-item measure (*I feel burned out*) also demonstrated acceptable communality (*h*^*2*^ = .71) and acceptable test-retest reliability at an 18-month lag (r_tt_ = .64/.54). West et al. [66] reported evidence of criterion validity of a single-item measure of emotional exhaustion extracted from the MBI across several samples of medical students.

As regards single-item measures of turnover intention, in spite of their extensive use, there has been no psychometric assessment of their reliability. Therefore, in addition to the existing evidence, in the present study, the quality of single items was checked on the basis of the previous research conducted on French firefighters [21; 61]. First, for each scale, the most highly loaded item was chosen. For SWLS-W and MBI, the item selection aligned with the previous research [61; 67]. The most general and clear item was extracted for the scale of turnover intention [68]. Second, the corrected item-total correlation was evaluated. Finally, the magnitude of associations with key outcomes were compared between single-item measures and full measures.

With regard to the aforementioned constraints, in order to balance measurement precision with respondent burden, a 4-point response format was selected. On the one hand, reliability gains plateau beyond four to five response categories, with limited incremental validity obtained from longer formats; on the other one, increasing the number of response options may impose additional cognitive demand on respondents required to differentiate between finely graded categories [34,35]. Therefore, all study variables were assessed using a 4-point Likert scale ranging from 1 (completely agree) to 4 (completely disagree).

1. The UMCS-22 items originally validated in Italian on a student sample [9] were translated into French by the bilingual expert panel (*N* = 3) and pretested by the target population panel (*N* = 10). The term “studies” was replaced by the term “volunteer firefighting activity” in French. Example item from the original UMCS-22: *This line of studies gives me immense personal satisfaction*. Same item in French adapted to VFF: *Je suis passionné(e) par mon activité de sapeur-pompier volontaire.*
2. One item from the French version of the Maslach Burnout Inventory (MBI) [69,70] assessing emotional exhaustion: *I feel emotionally drained from volunteer firefighting*. The proposed wording reflects the specificity of the French language, which does not have a strict equivalent for the verb “to burn out”. A similar item (worded in English as *I feel burned out*) was previously positively evaluated in a general English-speaking population [63]. This item showed high factor loading (*β* = .81) and high item-rest correlation (*r* = .83, *p* < .001) in a previous study, which supported its representativeness and reliability [67]. It was perceived as clear and non-ambiguous by the target population panel in the present study (4.8/5).
3. One item from the French version of the Satisfaction with Life Scale at Work (SWLS-W) [71,72]: *At present, I am satisfied with my activity of VFF.* The item was selected in accordance with previous recommendations [64; 65]. It had high factor loading (*β* = .83) and high item-rest correlation (*r* = .84, *p* < .001) in a previous study on French firefighters providing evidence for the single-item measure’s representativeness and reliability [61]. It was perceived as clear and non-ambiguous by the target population panel in the present study (5/5).
4. One item from the Scale of Intention to Leave in French [68]: *I have an intent to leave my Fire and Rescue Service in the next 6 months*. This item showed high factor loading (*β* = .89) and high item-rest correlation (*r* = .89, *p* < .001) in a previous study on French firefighters, therefore indicating that item can be considered representative of the overall construct and reliable [21]. It was perceived as clear and non-ambiguous by the target population panel in the present study (4.8/5). Control variables included age, gender, and length of service.

### Characteristics of the target population and study sample

Of the 253,000 firefighters in France, 198,900 are volunteers, accounting for 79% of the total [73]. Among them, 21% are female. The average age varies between 35 and 42 years depending on the category, while the average length of service is 11.9 years [74]. Volunteer commitments last for five renewable years, with compensation ranging from €8.36 to €12.58 per hour depending on rank [75]. Emergency rescue and traffic accident responses represent the majority of interventions (85%), while fires and wildfires account for 6%, and miscellaneous operations – for 9%, including industrial risks, pollution, and wildlife protection, are relatively infrequent [76].

With regard to the EU regulation of the use of personal data [77], the probabilistic sampling was not achievable. Hence, the coverage of the target population was assured via the nationwide invitation to take part in the Study hosted on LimeSurvey was advertised by the French National Federation of Firefighters during 6 weeks (19^th^ June – 17^th^ July 2023). and by a Fire and Rescue Departmental Service in the South of France during 6 weeks (16^th^ August – 27^th^ September 2023). The participation was not compensated. The withdrawal was allowed at any stage of the survey. The sociodemographic characteristics of the sample were further compared with the structure of the target population known from the aforementioned publicly available reports. Being an enlisted French VFF served an inclusion auto-selection criterion as it supposes a several-stage selection process, including the minimum age (16 years), legal residence in France, medical and physical aptitude screening, etc.

Out of 2,431 initiated questionnaires, 1,309 were fully completed. Of those 1,309 participants, 160 were excluded further to the outliers’ control via MFRM [42], thus leading to the sample reduction. The final sample was composed of 1,149 VFF with the following characteristics: 21.2% female respondents; mean age = 37.5 years, SD = 11.7; mean length of service = 15.4 years, SD = 11.0. The demographic characteristics of the sample were representative of the national profile [76].

## Results

The collected data were analyzed in Jamovi 2.3.3 to assess construct validity, reliability (internal consistency), convergent validity (construct level), and criterion validity of the French UMCS-22 in accordance with recommendations for scale development and validation [32; 78]. First, construct validity was evaluated using a measurement model (module SEM) of the French UMCS-22, focusing on the fit of the hypothesized seven-dimensional structure and to determine whether the items adequately represented the intended latent constructs. The measurement model for the French UMCS-22 met recommended cutoff values for model fit: *χ²*(df) = 339.554(188); CFI = .994; RMSEA = .038 (.036;.039); SRMR = .038). Measurement invariance was then tested across two genders and three age groups. The rationale for testing invariance across gender and age is detailed in the Method section (point 6a). All forms of measurement invariance were supported by the data between female and male participants (Table 1), and among the three age groups (Table 2). Regarding factor loadings (S1, Tables in S1 File), 5 items showed values below.70, but still over.65, which is considered acceptable. Therefore, the scores obtained from the French UMCS-22 adapted for VFF exhibited satisfactory *Construct Validity*.

**Table 1:** French UMCS-22 measurement invariance tests among male and female volunteer firefighters (*N*_*male*_ = 905; *N*_*female*_ = 244).

Model	*χ²*	df	CFI	RMSEA	SRMR
Configural invariance UMCS-22	384.333	376	.994	.040	.038
Metric invariance UMCS-22	461.443	391	.994	.043	.039
Scalar invariance UMCS-22	461.443	384	.994	.043	.039
Strict invariance UMCS-22	481.352	406	.994	.045	.038

*Nota bene:* Gender measurement invariance was tested across the groups of male and female participants. Estimation method: WLSMV.

**Table 2:** French UMCS-22 measurement invariance tests among age groups of volunteer firefighters (*N*_*16-24*_ = 194; *N*_*25-49*_ = 740; *N*_*50-65*_ = 214).

Model	*χ²*	df	CFI	RMSEA	SRMR
Configural invariance UMCS-22	397.831	504	.994	.042	.038
Metric invariance UMCS-22	613.437	532	.992	.051	.044
Scalar invariance UMCS-22	613.437	518	.992	.051	.044
Strict invariance UMCS-22	685.090	560	.991	.055	.045

*Nota bene:* Three age groups were established in accordance with INSEE (2024): 16–24; 25–49; 50–65 years. Estimation method: WLSMV.

Regarding *Reliability*, the dimensional scores demonstrated satisfactory internal consistency index ω-McDonald ranging between.75 and.86 (Table 3), thereby meeting the recommended thresholds [58,59]. The internal consistency estimates for the global score of the French UMCS-22 were both of.96.

**Table 3:** Descriptive statistics, Bayes factor correlations, and internal consistency indices for the French UMCS-22 adapted for French volunteer firefighters (*N* = 1,149).

N°	Variable	*M*	*SD*	1	2	3	4	5	6	7	8	9	10	11	12	13	14
1	Passion	3.20	.58	(.83/.83)													
2	Sacrifices	2.82	.71	.78	(.85/.86)												
3	Transcendent summons	2.94	.66	.69	.67	(.81/.81)											
4	Prosocial orientation	3.13	.54	.66	.64	.68	(.74/.75)										
5	Pervasiveness	2.97	.62	.75	.73	.70	.68	(.80/.81)									
6	Purposefulness	3.03	.62	.80	.72	.76	.72	.81	(.82/.83)								
7	Identity	3.12	.62	.80	.75	.77	.69	.78	.84	(.80/.81)							
8	Global UMCS-22 score	3.04	.58	.89	.87	.86	.81	.89	.92	.91	(.96/.96)						
9	Emotional exhaustion	1.80	.73	−.30	−.24	−.16	−.18	−.16	−.23	−.21	−.24	—					
10	Satisfaction with VFF	3.10	.67	.61	.55	.39	.44	.44	.50	.50	.56	−.34	—				
11	Turnover intention	1.43	.71	−.51	−.50	−.32	−.33	−.37	−.42	−.46	−.48	.38	−.55	—			
12	Age	37.48	11.65	−.22	−.17	−.15	−.16	−.20	−.17	−.18	−.20	.15	−.17	.19	—		
13	Length of service	15.43	11.06	−.19	−.13	−.11^*^	−.18	−.14	−.14	−.11^*^	−.16	.20	−.22	.22	.76	—	
14	Gender (1 = male)	—	—	.09^0^	.04^00^	.06^00^	.06^00^	.03^00^	.07^00^	08^00^	.07^00^	−.04^00^	.09^00^	−12	−.25	−.33	—

*Nota bene:* M – mean; SD – standard deviation. All variables are correlated with Bayes factors BF₁₀ > 100, except those marked with ”*”, which show BF₁₀ > 30, those marked with “00”, which show BF₁₀ < 1, and those marked with “0”, which show BF₁₀ < 10. In parenthesis are displayed (1) α-Chronbach and (2) ω-McDonald coefficients.

All the subscales of the French UMCS-22 showed acceptable *Convergent Validity* (AVE = .57 −.68) except for the subscale of *Prosocial orientation* (AVE = .49), which was close to the recommended threshold (AVE ≥ .50). Therefore, each subscale comprised items sharing a high proportion of common variance associated with the construct.

*Criterion Validity* of the French UMCS-22 scores was assessed via methodological triangulation, including Bayesian correlation (Table 3), Bayesian linear regression (Table 4), and modeling in latent variables via SEM (Table 5). To remind, the advantage of probabilistic statistics lies in the assessment of the strength of evidence in favor for both the research hypothesis (H1) and the null hypothesis (H0), with BF > 10 considered strong evidence, and the probability of inclusion into the model, with P > .80 considered high [38; 39]. These approaches also take into account sample characteristics, such as size and distribution, without requiring normally distributed data. In contrast, strict frequentist approaches generally assume normality and primarily focus on testing H1, without evaluating the probability of the evidence in favor of H0. With regard to directed versus nondirected relationships, regression and SEM analyses are currently preferred over correlational one [32,40]. Bayesian regression provides, in addition to the estimation of explained variance (R^2^) and effect size (*ß*), the probability of inclusion of the predictor into the model and the magnitude of Bayes factor supporting either H1 or H0, thereby enabling a more precise evaluation of predictor importance [39]. Current research in social and medical sciences increasingly adopts Bayesian approaches as alternative to traditional null hypothesis significance testing, emphasizing their ability to quantify evidence and avoid the limitations of *p*-values [41]. In this regard, Bayesian linear regression was identified as a primary method for assessing the criterion validity of the French UMCS-22, while Bayesian correlation served as a complementary descriptive approach, and SEM as a frequentist causal modeling alternative despite its reliance on *p*-values.

**Table 4:** Estimation of the effects of the calling dimensions of the French UMCS-22 onto satisfaction, emotional exhaustion and turnover intention in French volunteer firefighters via Bayesian linear regression (*N* = 1,149).

Predictor	Mean (Interval)	*SD*	P_inclusion_	BF_10_
*Criterion: Satisfaction, R*^*2*^ *= .40*				
Passion	.60 (.51; 70)	.04	1.00	4.904479906471974e + 23
Sacrifice	.22 (.14;.29)	.03	1.00	79761.49
Transcendent summons	−.13 (−.20; −.05)	.03	.96	25.33
Prosocial orientation	.14 (.05;.22)	.04	.81	4.20
Pervasiveness	−.12 (−.20; −.03)	.04	.81	4.24
*Criterion: Exhaustion, R*^*2*^ *= .10*				
Passion	−.51 (−.61; −.40)	.05	1.00	3077388095.41
Pervasiveness	.17 (.08;.27)	.04	1.00	23.28
*Criterion: Turnover intention, R*^*2*^ *= .31*				
Passion	−.39 (−.50; −.28)	.05	1.00	2.7730263862552263e + 50
Sacrifice	−.31 (−.40; −.23)	.04	1.00	166697136031.86
Transcendent summons	.19 (.11;.28)	.04	1.00	6650.99
Pervasiveness	.16 (.07;.26)	.05	1.00	10.73
Identity	−.25 (−.36; −.14)	.05	.96	2128.43
*Criterion: Satisfaction, R*^*2*^ *= .31*				
UMCS-22	.69 (.63;.75)	.03	1.00	3.354662331574079e + 91
*Criterion: Exhaustion, R*^*2*^ *= .06*				
UMCS-22	−.32 (−.40; −.25)	.04	1.00	24144451730660.48
*Criterion: Turnover intention, R*^*2*^ *= .23*				
UMCS-22	−.62 (−.69; −.55)	.03	1.00	5.20740908879819e + 62

*Nota bene*: P_inclusion_ – probability of inclusion with the data; BF_10_ – Bayes Factor supporting H1. Estimation was conducted with a Hyper-g prior distribution. The effects are derived from the posteriori summary of the best model for each criterion (Module JSQ, Jamovi 2.3.3).

**Table 5:** Assessment of the French UMCS-22 criterion validity via SEM (*N* = 1,149).

Predictor	*b*	SE	*β*	CI
*Outcome: Satisfaction (R*^*2*^ *= .38)*				
Passion	.99	.05	.62	[.57;.66]
*Outcome: Exhaustion (R*^*2*^ *= .14)*				
Passion	−1.26	.20	−.71	[-.92; -.51]
Pervasiveness	.67	.17	.47	[.24;.67]
*Outcome: Turnover intention (R*^*2*^ *= .27)*				
Passion	−.89	.06	−.51	[-.60; -.46]

*Nota bene*. *b* – non-standardized estimate; SE – standard error for non-standardized estimate; *ß* – standardized estimate; CI – 95% confidence interval for standardized estimate. Estimation method: WLSMV; bootstrap with 1,000 iterations. Model adjustment: *χ*^*2*^/df = 77.72/30; CFI = .994; TLI = .991; RMSEA = .037 (.027;.048); SRMR = .038.

First, correlations were calculated between the seven subscales and the global score of the French UMCS-22 and the three criterion variables. All correlations were supported with extreme evidence (BF_10_ > 100) except two (length of service and identity; length of service and transcendent summons) supported with strong evidence (BF_10_ < 30). The magnitude of correlations varied from weak to moderate for emotional exhaustion and moderate to large for job satisfaction and turnover intention. The global score of the French UMCS-22 was significantly correlated with all the study outcomes (Table 3).

Second, a series of Bayesian linear regressions was conducted (Table 4). The results indicated that some subscale scores of the French UMCS-22 were not predictive of the examined outcomes, while others demonstrated adverse effects. Specifically, *Purposefulness* did not show any significant effect. *Prosocial Orientation* was only predictive of satisfaction with the volunteering activity with moderate evidence (*ß* = .14, P_inclusion_ = .81, BF_10_ = 4.20). *Identity* affected exclusively turnover intention with extreme evidence (*ß* = −.25, P_inclusion_ = .96, BF_10_ = 2128.43). *Passion* consistently predicted all three outcomes, with a high probability of inclusion, a large effect size, and extreme evidence. (*ß* = −.39, P_inclusion_ = 1.00, BF_10_ = 2128.43), moderate for job satisfaction (*ß* = −.96, P_inclusion_ = .96, BF_10_ = 2128.43), and extreme for emotional exhaustion (*ß* = −.96, P_inclusion_ = .96, BF_10_ = 2128.43). *Sacrifice* was associated with both satisfaction and turnover intention with an extreme evidence and moderate effect size (*ß* = .22, P_inclusion_ = 1.00, BF_10_ = 79761.49). The most notable finding concerned the effects of *Transcendent Summons* and *Pervasiveness*. *Transcendent Summons* showed detrimental effects: of small magnitude and strong evidence on satisfaction with firefighting (*ß* = −.13, P_inclusion_ = .96, BF_10_ = 25.33) and of small magnitude and extreme evidence on turnover intention (*ß* = .22, P_inclusion_ = 1.00, BF_10_ = 6650.99). *Pervasiveness* exhibited aversive effects on all the three outcomes: of small magnitude and moderate evidence on satisfaction with firefighting (*ß* = −.12, P_inclusion_ = .81, BF_10_ = 4.20), of small magnitude and strong evidence on emotional exhaustion (*ß* = .17, P_inclusion_ = 1.00, BF_10_ = 23.28), and of small magnitude and strong evidence on turnover intention (*ß* = .16, P_inclusion_ = 1.00, BF_10_ = 10.73). It is recommended, in the interpretations of the results of Bayesian estimations, to favor predictors with strong (BF_10_ > 10) to extreme (BF_10_ > 100) evidence over those with moderate evidence [79]. To summarize the French UMCS-22 dimensions that predict the outcomes with extreme evidence, they include: *Passion* and *Sacrifice* for job satisfaction, *Passion* for emotional exhaustion, and *Passion*, *Sacrifice, Identity,* and *Transcendent Summons* for turnover intention*.* As regards the global score of the French UMCS-22, it predicted the outcomes with extreme evidence (BF > 100) and moderate to strong magnitude. At the same time, it explained lower variance in each outcome as compared with the French UMCS-22 dimensions. For instance, the scores of five French UMCS-22 dimensions – *Passion, Sacrifice, Transcendent Summons, Pervasiveness,* and *Identity* – explained 31% of the variance in turnover intention, whereas the global score of the French UMCS-22 accounted for 23% (Table 4).

**Fig 1:**
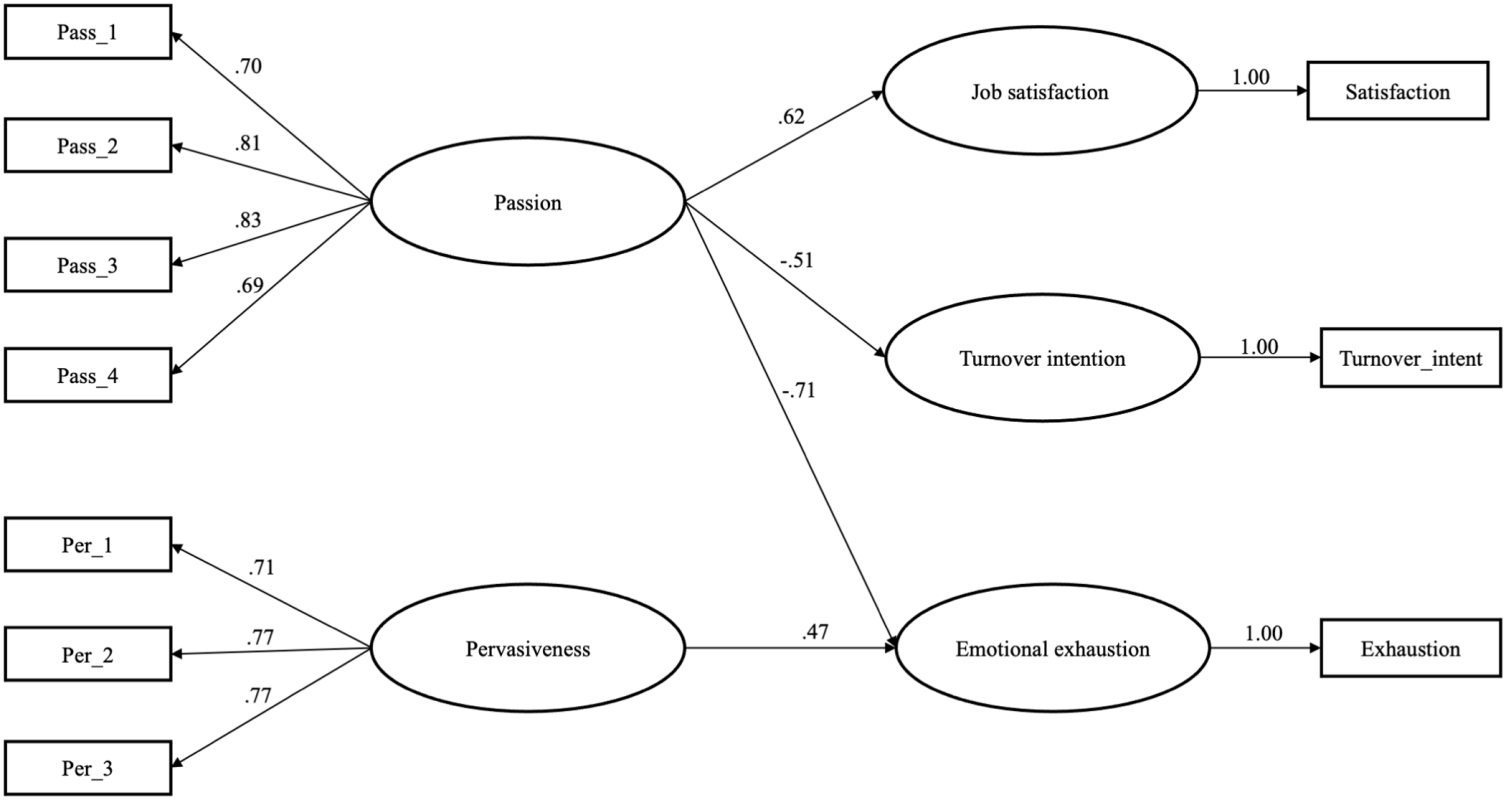
Results of the assessment of the French UMCS-22 criterion validity via SEM (N = 1,149): passion and pervasiveness as predictors of job satisfaction, emotional exhaustion, and turnover intention in French volunteer firefighters. *Nota bene.* Model adjustment (WLSMV; bootstrap with 1,000 iterations): *χ*^2^/df = 77.72/30; CFI = .994; TLI = .991; RMSEA = .037 (.027;.048); SRMR = .038. Standardized path coefficients ß are indicated.

In addition to Bayesian regression analysis, a structural equation modeling (SEM) approach was used as frequentist alternative for latent variable estimation (Table 5). The estimation was conducted via a first-order latent factor model, where the French UMCS-22 dimensions and outcome variables were specified as latent variables represented by the corresponding manifest indicators (items) (for more details on protocol items, see Supplementary material S1, Tables in S1 File and data https://osf.io/skzwt/files/hn46w). The technique WLSMV (weighted least squares means and variances adjusted) was used with regard to the 4-point format of the response scale. The robustness of the effects was evaluated via a Bootstrap technique (1,000 iterations). Seven facets of the UMCS-22 were specified as predictors, whereas job satisfaction, emotional exhaustion, and turnover intention were specified as outcome variables. All predictors were allowed to covary freely, whereas the residuals of the outcome variables were constrained not to covary. The measurement model, including seven dimensions of the French UMCS-22 and three outcomes, showed satisfactory fit: *χ*^*2*^/df = 470.796/233; p < .01; CFI = .993; TLI = .991; RMSEA = .040 (.038;.042); SRMR = .040. Although the structural model containing all seven facets of the French UMCS-22 exhibited satisfactory fit (*χ*^*2*^/df = 470.818/235; p < .01; CFI = .993; TLI = .991; RMSEA = .030 (.026;.033); SRMR = .040), several paths were non-significant. Therefore, the French UMCS-22 facets that had no significant effects on the three outcome variables were removed from the model. The structural model including only significant predictors (*Passion* and *Pervasiveness*) remained satisfactory: *χ*^*2*^/df = 77.72/30; CFI = .994; TLI = .991; RMSEA = .037 (.027;.048); SRMR = .038 (Fig 1, Table 5). According to this model, *Passion* was the only significant predictor for job satisfaction and turnover intention, whereas emotional exhaustion was predicted by both *Passion* and *Pervasiveness.* Across all three outcomes, the effect of *Passion* was large (*ß* ≥ .50), while the effect of *Pervasiveness* on exhaustion was close to large (*ß* = .47).

The three methods of estimation provide converging evidence. Correlational analysis establishes baseline associations but does not estimate the unique contribution of each dimension of the French UMCS-22. Indeed, all the French UMCS-22 dimensions correlate positively with satisfaction and negatively with emotional exhaustion and turnover intention. Bayesian regression quantifies the strength of evidence for competing predictive models while offering flexibility with respect to distributional assumptions [38,39]. SEM models the relationships at the latent level, explicitly accounting for measurement error [40]. The limitation of Bayesian correlational analysis lies in its inability to evaluate the strength of evidence for each predictor. The limitation of SEM, as implemented in Jamovi 2.3.3, relates to its reliance on *p*-values to evaluate the significance of predictors. Although the relationships between *Passion* and the outcomes are supported by three methods, as well as the relationship between *Pervasiveness* and emotional exhaustion, the application of the ATOM principle [41] supports focusing on the French UMCS-22 dimensions that are associated with strong to extreme evidence (Table 4) as relevant predictors of job satisfaction, emotional exhaustion, and turnover intention in VFF.

In addition to the primary purpose of this validation study, associations of calling dimensions with gender and age were examined via Bayesian *T-test* and *ANOVA* respectively (Tables 6,7). Following conventional guidelines, Bayes factor greater than 3.00 was interpreted as providing moderate evidence for the hypothesis support, whereas Bayes factor close to 1.00 was associated with a weak to anecdotal support [38,39]. The analysis revealed moderate evidence for gender difference only for *Passion* (BF_10_ = 7.63). In contrast, the results favored null hypothesis with moderate evidence for *Pervasiveness* (BF_01_ = 7.93) and *Sacrifice* (BF_01_ = 4.77), suggesting no meaningful gender difference in these dimensions. Specifically, female VFF reported higher levels of *Passion* than their male counterparts. *Pervasiveness* and *Sacrifice* showed comparable levels across genders. No clear evidence was found in favor of either the null hypothesis (H0) or the alternative hypothesis (H1) regarding gender differences in the global French UMCS-22 score (Table 6).

**Table 6:** Bayes factor *T*-test results for gender differences in the French UMCS-22 dimensions (N_male_ = 905; N_female_ = 244).

N°	Variable	Mean (Standard Deviation)		BF_10_, model	BF_01_, model	Error %, model
		Male	Female			
1	Passion	3.17 (.60)	3.30 (.52)	7.63	.13	.00
2	Sacrifices	2.80 (.71)	2.87 (.67)	.21	4.77	.09
3	Transcendent summons	2.92 (.67)	3.02 (.61)	.51	1.96	.04
4	Prosocial orientation	3.11 (.54)	3.19 (50)	.69	1.45	.03
5	Pervasiveness	2.96 (.63)	3.01 (.60)	.13	7.93	.15
6	Purposefulness	3.01 (.62)	3.11 (.58)	1.23	.81	.02
7	Identity	3.10 (.62)	3.21 (.58)	1.99	.50	.01
8	Global UMCS-22 score	3.36 (.45)	2.96 (.53)	1.09	.92	.02

*Nota bene.* BF_10_ – Bayes factor supporting H1 over H0; BF_01_ – Bayes factor supporting H0 over H1.

**Table 7:** Bayes factor ANOVA results for age group-related differences in the French UMCS-22 dimensions (N_16-24_ = 194; N_25-49_ = 740; N_50-65_ = 214).

N°	Variable	Mean (Standard Deviation)			Post-hoc group comparison, error %			BF_10_, model	Probability of inclusion with data	Error %, model
		1	2	3	1-2	1-3	2-3			
1	Passion	3.59 (.41)	3.12 (.58)	3.13 (.60)	.00	.00	.21	545028938111917555712.00	1.00	.02
2	Sacrifices	3.19 (.58)	2.74 (.70)	2.76 (.72)	.00	.00	.20	925871323168.12	1.00	.03
3	Transcendent summons	3.20 (.64)	2.91 (.63)	2.84 (.72)	.00	.00	.09	93609.93	1.00	.02
4	Prosocial orientation	3.40 (.47)	3.07 (53)	3.11 (.55)	.00	.00	.14	59999326354.06	1.00	.03
5	Pervasiveness	3.34 (.52)	2.90 (.62)	2.89 (.61)	.00	.00	.21	7513650554108009.00	1.00	.02
6	Purposefulness	3.36 (.54)	2.96 (.61)	2.98 (.60)	.00	.00	.19	6479069453916.32	1.00	.02
7	Identity	3.45 (.50)	3.05 (.62)	3.05 (.60)	.00	.00	.21	101448581162.96	1.00	.02
8	Global UMCS-22 score	3.36 (.45)	2.96 (.53)	2.96 (.55)	.00	.00	.21	47511662243319928.00	1.00	.02

*Nota bene*. Age cohorts are defined in accordance with the taxonomy by French Institute of Statistics and Economics Research (INSEE, 2025) as follows: 1–16–24 years, 2–25–49 years, 3–50–65 years. BF_10_ – Bayes factor supporting H1 over H0.

Bayesian ANOVA revealed extreme evidence for the age difference across all the French UMCS-22 dimensions as well as for the global score (BF₁₀ > 100). Post-hoc comparisons indicated that the 16–24-year-old group reported higher levels of calling than both the 25–49 and 50–65 groups, whereas evidence for differences between the two older cohorts was weak to anecdotal. The probability of inclusion for the age factor was equal to 1.00 in all models, indicating that age meaningfully contributed to explaining variance in calling (Table 7).

## Discussion

This study examined the psychometric properties of the French UMCS-22 adapted for stipended volunteer firefighters (VFF). They included *Construct Validity, Convergent Validity,* *Reliability,* and *Criterion Validity.* Due to the limitations in the research design discussed in the Method section, discriminant, divergent, predictive forms of validity, as well as Test-retest reliability were not evaluated. The assessment of the French UMCS-22 was conducted at both the dimensional and global levels. Theoretical discussions of the relationship between modern and neoclassical facets of calling, as well as the assumption of the potential dark side of calling guided the decision to focus on the 7 dimensions of French UMCS-22 [4,8,9,15]. It is important to note that the items of the present version of the UMCS-22 were worded to respect the original meaning, while specifying firefighting activity as a target of calling. Given that calling is a construct targeted toward a specific domain or activity, the wording of the items should be context-specific [6]. In this regard, French items of the current version may be adapted to other populations if the target occupation is specified.

*Construct validity* of the dimensional and global scores of the French UMCS-22 was demonstrated thanks to the satisfactory factorial solution as well as thanks to gender-based and age-based strict measurement invariance, thus informing researchers and practitioners of the robustness of the scale and possibility to compare age and gender groups. The sizes of internal consistency indices of the French UMCS-22 scores were consistent with the conventional guidelines, therefore informing of the reliability of the dimensional and global scores of the French UMCS-22. In addition, all the dimensional scores demonstrated satisfactory *Convergent Validity*. With regard to the criterion validity of the French UMCS-22 scores, it was assessed via the methodological triangulation, including Bayesian correlation, Bayesian linear regression, and structural equation modeling (SEM) in latent variables. While SEM estimates relations among latent dimensions of the French UMCS-22, including measurement error [40], it is based on the *p*-value test and therefore, presents certain limitations in terms of hypotheses testing [38,39,41]. Bayesian regression evaluates the extent to which observed scores contribute to predictive models and quantifies evidence for their inclusion [38,39]. Therefore, in spite of divergent results, those obtained via Bayesian regression, with strong to extreme evidence, are favored in the present research.

The global score of the UMCS-22 was confirmed as predictor of job satisfaction, emotional exhaustion, and turnover intention in VFF, which aligns with the existing literature [8–10,13,15,80]. The dimensional analysis of the French UMCS-22 scores enabled a more nuanced analysis of the aspects of calling associated with the modern and neoclassical approaches. Indeed, Bayesian linear regression was useful in distinguishing calling dimensions either as protective or risk factors. Thus, according to the results of SEM, *Passion* appears to be a powerful resource associated with job satisfaction and preventing VFF from emotional exhaustion and turnover intention, while *Pervasiveness* contributes to emotional exhaustion. Bayesian linear regression led to identify more UMSC-22 dimensions as predictive of the studied outcomes. Thus, *Transcendent Summons* and *Pervasiveness* did not function as protective psychological resources; rather, they emerged as risk factors associated with decreased satisfaction, increased emotional exhaustion, and higher turnover intention in VFF. These findings support the idea of the dual-facet nature of calling [3,15]. *Purposefulness* had none, either positive or negative, effect onto the three examined outcomes in French VFF.

In addition to the main purpose, the present study examined gender- and age-related differences in calling among French VFF. A moderate difference was observed in *Passion*, which is consistent with several findings, according to which women demonstrate higher scores of calling [1]. Otherwise, gender differences were largely unsupported, indicating that calling is experienced similarly by male and female VFF. This finding is consistent with theoretical views of calling as an identity-based construct that is relatively independent of demographic characteristics, thereby supporting the generalizability of the UMCS-22 across genders. On the contrary, the study revealed marked age-related differences across all French UMCS-22 dimensions, with participants aged 16–24 reporting systematically higher levels of calling than those in the 25–49 and 50–65 age groups. Bayesian analysis provided decisive evidence in favor for the distinction between emerging adults and older VFF, indicating greater salience of calling in the beginning of the vocational engagement that declines though life-span. This finding is consistent with the meta-analytic conclusions of Dobrow et al. [8] and the discussion of Vianello et al. [9] emphasizing the dynamic nature of calling associated with the identity-related mechanisms. Calling may be experienced more intensely during the early stages of occupational trajectory, when individuals question and define their vocational perspectives. A subsequent decrease in its intensity may reflect more stable relationship with one’s occupation rather than a diminution of its significance.

### Theoretical implications

The UMCS-22 [9] was developed to encompass all dimensions of calling as conceptualized within two concurrent theoretical frameworks, the neoclassical and modern approaches. The rationale of the neoclassical perspective emphasizes external orientation and social utility of the occupations. Accordingly, this perspective is associated with *Transcendent Summons, Sacrifice,* and *Prosocial Orientation* [e.g., 1]. In contrast, the modern approach conceptualizes calling as internally driven by the pursuit of self-actualization, and includes the dimensions of *Passion, Identity,* and *Pervasiveness* [e.g., 8]. *Purposefulness* seems to be a transversal dimension present in both approaches [1,6–8,10]. For Bunderson and Thompson [7], the modern conceptualization of calling reflects a matter of personal choice, while the neoclassical one is grounded in the notions of duty and destiny and, therefore, is expected to be characterized by a higher intensity and strength.

Two issues have been highlighted in the recent literature with regard to the dimensions of calling: (1) whether the facets relative to the neoclassical and modern approached show greater relative prominence; (2) which dimensions are associated with the potential dark side of calling [1,3,7–9,15]. Thus, Dobrow et al. [8], who compared two broad categories of calling, externally and internally driven ones, reported that job satisfaction was more strongly associated with externally driven (modern) forms of calling, whereas meaningfulness of work and psychological well-being was more dependent on internally oriented (neoclassical) aspects of calling. Hagmaier and Abele [10] found, in German and North American samples, that the person-environment fit dimension, reflecting self-accomplishment and passion, positively predicted job satisfaction; when associated with the transcendent dimension, functioned as a protective factor against job exhaustion. Vianello et al. [9], who designed the UMCS-22 in Italian, identified several potentially detrimental outcomes of calling in their scale validation study, associated with *Identity, Transcendent summons* and *Pervasiveness*. These nuanced findings are among very few attempts to distinguish empirically the consequences of different aspects of calling. Thus, *Prosocial orientation* and *Identity* diminish satisfaction with studies, while *Transcendent summons* and *Pervasiveness* exhibit negative, although nonsignificant, effects on satisfaction and intention to pursue studies in Italian students [9].

In the present study, a comparison of the effects of calling dimensions, drawn from both theoretical approaches, highlights *Passion* (modern approach) and *Sacrifice* (neoclassical approach) as those exerting the most substantial positive effects on job satisfaction, emotional exhaustion and turnover intention in VFF. On the contrary, *Pervasiveness* (modern approach) and *Transcendent Summons* (neoclassical approach) were associated with negative outcomes, such as decreasing satisfaction with firefighting and increasing emotional exhausting and turnover propension. Given that *Prosocial Orientation* (neoclassical approach) shows a small positive association with job satisfaction with moderate evidence, its role should be clarified and confirmed in the future research. *Identity* (modern approach) displays a moderate negative association with turnover intention. *Purposefulness* score (neoclassical and modern approach) did not predict any outcome either via Bayesian linear regression or via SEM. In this respect, the results of the present study contribute to the identification of the calling aspects that may be detrimental for the work and para-work domains. Specifically, *Transcendent Summons* and *Pervasiveness* may function as psychosocial risk factors, diminishing satisfaction with volunteering and increasing emotional exhaustion and turnover intention. These dimensions may conceptually converge with the construct of obsessive passion described by Vallerand et al. [81], thereby offering new avenues for understanding the nature of calling, for example, in the form of the theoretical bridge between the literatures on calling and passion. Previous research has highlighted the adverse effects of extreme forms of calling, which may lead to workaholism, addiction to work and, further, to the occupational health impairments, including sleep and affect disorders [e.g., 3, 8]. Both, *Transcendent Summons* and *Pervasiveness* involve a perceived omnipresence of the vocational role and sustained cognitive rumination, which foster the development of maladaptive potentially addictive domain-related cognitions and behaviors.

Both the neoclassical and modern approaches to calling seem to be informative with regard to its positive and negative outcomes. However, *Passion*, a dimension incarnating the modern approach demonstrates the largest effects among all the French UMCS-22 facets, which aligns with the findings of Vianello et al. [9]. The comparison of the detrimental and beneficial effects of the calling dimensions in French VFF and in the original validation study [9] suggests that *Passion* and *Sacrifice* may reflect the more adaptive bright side of calling, whereas *Transcendent Summons* and *Pervasiveness* may represent its dark side [3,7,8]. The functioning of the remaining calling dimensions requires further investigation in other populations to clarify and generalize their roles.

### Practical implications

The French UMCS-22 can be employed as a diagnostic tool for VFF and volunteers in general. The informed use of the French UMCS-22 may support individuals in identifying the most salient facets of calling towards volunteer activities in ways that foster their well-being and self-actualization. In line with the findings of the present validation study, *Passion* and *Sacrifice* should be considered as key indicators of well-being and satisfaction in volunteer work, while *Pervasiveness* and *Transcendent Summons* should be taken as a psychosocial risk factors. In this regard, psychologists and managers should address potential adverse outcomes of calling in volunteer workers, such as emotional exhaustion and turnover intention.

At the organizational level, calling should be fully recognized by institutions relying on stipended volunteers, such as fire and rescue services, emergency health and disaster management services, as a valuable psychological resource that enhances organizational embeddedness while mitigating risks of health impairments. From this perspective, the French UMCS-22, thanks to its multifaceted nature, constitutes a promising diagnostic instrument for occupational health prevention. Indeed, high scores on *Transcendent Summons* and *Pervasiveness* should inform organizations about potential risk of work exhaustion and further turnover.

### Limitations and directions for future research

The present validation study does not come without limitations. First, due to the restricted access to the VFF personal data, the study adopted a cross-sectional design, which did not allow for the assessment of test-retest reliability and predictive validity, nor for observing the changes in calling associated with socio-demographic (e.g., age) and organizational factors. Second, the target population is characterized by high levels of calling compared with the general population [21,22,24–26,61]. Third, given constraints related to participant availability, several single-item measures were used in the protocol, which may constitute a potential source of bias. Fourth, due to the same constraint, divergent and discriminant forms of validity, requiring the inclusion of the additional instruments into the protocol, were not assessed. Some of these limitations were partially mitigated by the use of Bayesian estimation techniques, which enabled the comparison of competing predictive models, including those associated with null hypothesis, thereby providing a more nuanced analytical approach [38,39].

Several future research directions are envisaged to both address the limitations of the present study and advance research on calling and its complex nature. The evaluation of the construct validity of the French UMCS-22 could be strengthened by examining its cross-cultural measurement invariance, for example, via a comparative study of VFF in different countries. A longitudinal design would allow for a more robust appraisal of measurement invariance, predictive validity, and test-retest reliability of the French UMCS-22. In addition, the discriminant validity of the French UMCS-22 should be evaluated in relation to the instruments assessing passion and meaningful work. Convergent validity should be examined with the help of the established calling scales. In line with the previous recommendations [8,9,15] and the findings of this study, it would be pertinent to compare the stability of the seven facets of calling. Future research should also extend the psychometric evaluation of the French UMCS-22 to other occupational groups to better understand the role of calling dimensions that did not demonstrate strong effects on the outcomes. Overall, future research should adhere to the principle of estimation triangulation, thereby enabling more cautious interpretations of *p*-values [41,79].

## Conclusion

This research confirms that the scores of the French UMCS-22 adapted for VFF exhibit the expected psychometric properties, including construct validity, convergent validity, reliability, and criterion validity. The findings suggest that calling may function both as a resource through passion, sacrifice, and identity, as well as a potential risk factor through transcendent summons and pervasiveness. In this regard, the French UMCS-22 has a utility for anticipating and preventing health impairments and organizational withdrawal in the population targeted by the validation study. Future research adopting a longitudinal design and the principle of estimation triangulation will shed more light on current unaddressed issues, including cross-cultural measurement invariance, discriminant and convergent validity, and test-retest reliability of the French UMCS-22.

## Supporting information

S1 File Supplementary material. (PDF)

S2 File Data_French_UMCS-22. Codebook and data.
(ZIP)
