# Supplementary material for: Validation and utility of the French version of the Unified Multidimensional Calling Scale (UMCS-22) for stipended volunteer firefighters
Source: PLoS One. 2026 May 28;21(5):e0350184. doi: 10.1371/journal.pone.0350184 (PMC13218494; doi:10.1371/journal.pone.0350184)
Supplement: S1 File — (PDF) [file pone.0350184.s001.pdf]

Validation and Utility of the French version of the Unified Multidimensional Calling Scale (UMCS-22) for Stipended Volunteer Firefighters

Supplementary material

Table 1 SM.

English and French wordings of the UMCS-22.

Code	English wording for students (Vianello et al., 2018)	French wording for volunteer firefighters
Pass_1	I am passionate about what I am studying.	Je suis passionné(e) par mon activité de SPV.
Pass_2	I enjoy what I study more than anything else.	Mon activité de SPV me plaît plus que toute autre chose.
Pass_3	This line of studies gives me immense personal satisfaction.	Mon activité de SPV me donne une énorme satisfaction.
Pass_4	My current line of studies gives me exciting and deeply gratifying experiences.	Mon activité de SPV me permet de vivre des expériences intenses et émotionnelles.
Sac_1	I would keep studying this subject even in the face of severe obstacles.	Je continuerais à être SPV malgré d'importants obstacles.
Sac_2	I can give up many things to keep studying this subject.	Je peux renoncer à beaucoup de choses afin de préserver mon engagement de SPV.
Sac_3	I can deal with many sacrifices to keep studying this subject.	Je suis disposé(e) à faire de grands sacrifices pour maintenir mon activité de SPV.
TrS_1	I am pursuing this line of study because I believe I have been called to do so.	Je poursuis mon activité de SPV parce que je pense être « appelé(e) » à l'exercer.
TrS_2	I have been called by something beyond myself to pursue my current line of study.	J'ai été « appelé(e) » à m'engager dans l'activité de SPV par quelque chose qui va au-delà de moi-même.
TrS_3	I believe that I have been called to pursue my current line of study.	Je crois que j'ai été « appelé(e) » à devenir SPV.
Pro_1	The most important part of my future work is helping others to meet their needs.	En tant que SPV, je cherche à contribuer à la satisfaction des besoins des autres.
Pro_2	I always consider how beneficial my work will be to others.	Je cherche toujours à rendre mon activité de SPV bénéfique pour les autres.

Pro_3	Making a difference for others is my primary motivation in my academic and professional career.	Mon objectif principal en tant que SPV est d’améliorer la vie des autres
Per_1	Even when I am not studying, I often think about my courses.	Je pense à mon activité de SPV même en dehors des gardes/astreintes
Per_2	My current line of study is always on my mind.	Mon activité de SPV est toujours dans mes pensées.
Per_3	My days would be less meaningful if I was not involved in these studies.	Mes journées ont du sens grâce à mon activité de SPV.
Pur_1	I see my academic and professional career as a path to purpose in life.	Je vois mon activité de SPV comme un mode d’accomplissement de soi.
Pur_2	My academic and professional career is important to give meaning to my life.	Mon activité de SPV est importante, car elle apporte du sens à ma vie.
Pur_3	My academic and professional career helps me live out my life’s purpose.	Mon activité de SPV me permet de réaliser la mission de ma vie.
Ide_1	What I study will always be part of my life.	Mon activité de SPV fera toujours partie de ma vie.
Ide_2	What I study is part of who I am.	Mon activité de SPV fait partie de moi.
Ide_3	What I study is part of my destiny.	Mon activité de SPV fait partie de mon destin.

Table 2 SM.

French UMCS-22 measurement invariance across the samples (S1=888; S2=421).

Model	X^2	df	CFI	RMSEA	SRMR
Configural invariance UMCS-22	2110.356	332	.906	.088	.046
Metric invariance UMCS-22	2141.792	352	.906	.086	.051
Scalar invariance UMCS-22	2291.706	345	.898	.091	.054
Strict invariance UMCS-21	2129.277	343	.906	.087	.046

Table 3 SM.

Factors loadings of the original and French UMCS-22.

Dimension of the UMCS-22	Item code	Factor loadings of the original UMCS-22 (Vianello et al., 2018)	Factor loadings of the French UMCS-22
Passion	Pass_1	.80	.66
	Pass_2	.84	.82
	Pass_3	.82	.80
	Pass_4	.77	.68
Sacrifice	Sac_1	.93	.77
	Sac_2	.92	.84
	Sac_3	.91	.84
Transcendental summons	TrS_1	.92	.73
	TrS_2	.92	.76
	TrS_3	.92	.80
Prosocial orientation	Pro_1	.80	.68
	Pro_2	.82	.78
	Pro_3	.82	.79
Pervasiveness	Per_1	.84	.77
	Per_2	.93	.91
	Per_3	.28	.97
Purposefulness	Pur_1	.77	.73
	Pur_2	.70	.81
	Pur_3	.73	.79
Identity	Ide_1	.80	.75
	Ide_2	.87	.77
	Ide_3	.65	.76

Nota bene. UMCS - Unified Model Calling Scale. Measurement model was evaluated in Jamovi 2.3.3; module SEM, maximum likelihood estimation; factor loadings correspond to the standardized coefficients ß.
