# Supplementary material for: Validation and utility of the French version of the Unified Multidimensional Calling Scale (UMCS-22) for stipended volunteer firefighters
Source: PLoS One. 2026 May 28;21(5):e0350184. doi: 10.1371/journal.pone.0350184 (PMC13218494; doi:10.1371/journal.pone.0350184)
Supplement: S2 File — Codebook and data. (ZIP) [file pone.0350184.s002.zip › Supporting information/S1_File.pdf]

Validation and Utility of the French version of the Unified Multidimensional Calling Scale (UMCS-22) for Stipended Volunteer Firefighters

Supplementary material

Table 1 SM.

English and French wordings of the UMCS-22.

Code	English wording for students (Vianello et al., 2018)	English wording for volunteer firefighters (Burakova, 2026)
	I am passionate about what I am studying.	I am passionate about my activity as a volunteer firefighter (VFF).
Pass_2	I enjoy what I study more than anything else.	My activity of VFF pleases me more than anything else.
Pass_3	This line of studies gives me immense personal satisfaction.	My activity of VFF gives me immense personal satisfaction.
Pass_4	My current line of studies gives me exciting and deeply gratifying experiences.	My activity of VFF allows me to experience exciting and deeply gratifying experiences.
Sac_1	I would keep studying this subject even in the face of severe obstacles.	I would keep being a VFF even in the face of severe obstacles.
Sac_2	I can give up many things to keep studying this subject.	I can give up many to keep being a VFF.
Sac_3	I can deal with many sacrifices to keep studying this subject.	I can deal with many sacrifices to keep my activity of VFF.
TrS_1	I am pursuing this line of study because I believe I have been called to do so.	I pursue my activity VFF because I believe I was called to do so.
TrS_2	I have been called by something beyond myself to pursue my current line of study.	I have been called by something beyond myself to engage as VFF.
TrS_3	I believe that I have been called to pursue my current line of study.	I believe that I have been called to become a VFF.
Pro_1	The most important part of my future work is helping others to meet their needs.	As a VFF, I seek to contribute to helping others to meet their needs.
Pro_2	I always consider how beneficial my work will be to others.	I always consider how beneficial my activity of VFF is beneficial to others.
Pro_3	Making a difference for others is my primary motivation in my academic and professional career.	My main goal as a VFF is to improve the lives of others.

Code	English wording for students (Vianello et al., 2018)	English wording for volunteer firefighters (Burakova, 2026)
Per_1	Even when I am not studying, I often think about my courses.	I think about my activity of VFF even outside of duty/on-call periods.
Per_2	My current line of study is always on my mind.	My activity of VFF is always on my mind.
Per_3	My days would be less meaningful if I was not involved in these studies.	My days have meaning thanks to my activity of VFF.
Pur_1	I see my academic and professional career as a path to purpose in life.	I see my activity of VFF as a path to purpose in life.
Pur_2	My academic and professional career is important to give meaning to my life.	My activity of VFF is important to give meaning to my life.
Pur_3	My academic and professional career helps me live out my life's purpose.	My activity of VFF allows me to live out my life's purpose.
Ide_1	What I study will always be part of my life.	My activity of VFF will always be part of my life.
Ide_2	What I study is part of who I am.	My activity of VFF is part of who I am.
Ide_3	What I study is part of my destiny.	My activity of VFF is part of my destiny.

Nota bene. VFF - volunteer firefighter.

Table 2 SM.

French UMCS-22 measurement invariance across the samples (*S1* = 888; *S2* = 421).

Model	$\chi 2$	df	CFI	RMSEA	SRMR
Configural invariance UMCS-22	2110.356	332	.906	.088	.046
Metric invariance UMCS-22	2141.792	352	.906	.086	.051
Scalar invariance UMCS-22	2291.706	345	.898	.091	.054
Strict invariance UMCS-21	2129.277	343	.906	.087	.046

Table 3 SM.

Factors loadings of the original and French UMCS-22.

Dimension of the UMCS-22	Item code	Factor loadings of the original UMCS-22 (Vianello et al., 2018)	Factor loadings of the French UMCS-22 (Burakova, 2026)
Passion	Pass_1	.80	.66
	Pass_2	.84	.82
	Pass_3	.82	.80
	Pass_4	.77	.68
Sacrifice	Sac_1	.93	.77
	Sac_2	.92	.84
	Sac_3	.91	.84
Transcendental summons	TrS_1	.92	.73
	TrS_2	.92	.76
	TrS_3	.92	.80
Prosocial orientation	Pro_1	.80	.68
	Pro_2	.82	.78
	Pro_3	.82	.79
Pervasiveness	Per_1	.84	.77
	Per_2	.93	.91
	Per_3	.28	.97
Purposefulness	Pur_1	.77	.73
	Pur_2	.70	.81
	Pur_3	.73	.79
Identity	Ide_1	.80	.75
	Ide_2	.87	.77
	Ide_3	.65	.76

Nota bene. UMCS - Unified Model Calling Scale. Measurement model was evaluated in Jamovi 2.3.3; module SEM, maximum likelihood estimation; factor loadings correspond to the standardized coefficients *ß*.
